# Of Mice and Men, Redux: Modern Challenges in β Cell Gene Targeting

**DOI:** 10.1210/endocr/bqaa078

**Published:** 2020-05-18

**Authors:** Jennifer L Estall, Robert A Screaton

**Affiliations:** 1 Institut de recherches cliniques de Montréal, Montreal, Quebec, Canada; 2 Department of Biochemistry, Sunnybrook Research Institute and University of Toronto, Toronto, Canada

**Keywords:** β cell knockout, Cre-recombinase, gene targeting, promoter methylation, Ins1-Cre

The topic of which Cre-recombinase driver to use for gene targeting in pancreatic β cells comes up often around the bar at conferences. And these conversations last a long time. Challenges with the most commonly used tool for targeted gene recombination (Cre/Lox) plague the β cell field. With more than 20 lines generated to target these specialized endocrine cells, choice was never the problem.

Struggles began 15 years ago, when one strain of transgenic mice using the popular rat *Ins2* promoter to drive Cre-recombinase (*RIP*-Cre^25Mgn^) was shown to be glucose-intolerant, even in the absence of a floxed gene target (1). Others noticed similar effects, varying with line, background strain, and facility, but this raised sufficient concern that researchers began to avoid *RIP*-Cre lines. Popular alternatives included pancreatic and duodenal homeobox (*Pdx1*)-Cre lines, which drive expression in the endocrine cells of adult mice when used in conjunction with an inducible *Cre* transgene. However, experiments with reporter mice demonstrate these Cre lines, along with other popular *RIP* (*Ins2*)-driven lines, have significant Cre expression in areas of the digestive and central nervous systems (2). These data still fuel heated debate as to whether recombination at easily accessible reporter loci truly reflects efficiency of recombination at all floxed alleles. Frustrated by doubt, many researchers turned their backs on both the *Pdx1* and *Ins2* promoters. Next up was the murine *Ins1* gene promoter (MIP), which turns out to be much more specific to β cells (2, 3). This led to generation of *MIP*-Cre/ERT^1Lphi^ mice, a tamoxifen-inducible Cre line with the much-desired specificity and high recombination efficiency the field desperately needed (2, 3).

But the mystery as to why some β cell–targeted mouse lines have phenotypes independent of a floxed allele remained unsolved. An answer soon came, bringing with it another major hurdle with implications now reaching beyond islet biology. It was shown that the human growth hormone (hGH) minigene, commonly added to constructs for its polyA tail and introns that increase transcription efficiency, can also express bioactive growth hormone (4). Expression of ectopic hGH in β cells is not without consequences: *Pdx1*-cre1^Late^ mice display local activation of the prolactin receptor, impaired glucose-stimulate insulin secretion, increased β cell mass, and high insulin expression. This discovery finally shed light on the mysterious glucose intolerance phenotypes in some β cell targeting lines, but created a disaster for interpretation of data generated with the new *MIP*-CreERT^1Lphi^ mouse (which expresses the hGH minigene) (5). Although glucose homeostasis is less affected (5) in this line, mice have increased β cell mass (6) and reduced response to streptozocin, a toxin commonly used to model β cell insufficiency (5).

## Back to square one.

Soon came 2 new mouse strains: 1 driving constitutive Cre expression in β cells (*Ins1*-Cre^Thor^) and the other a tamoxifen-inducible Cre expresser (*Ins1*-CreERT^Thor^) (7), both using a knockin approach to the *Ins1* gene locus and eliminating the troublesome hGH minigene. Given that mice carry 4 copies of the insulin gene, disruption of one *Ins1* locus is thought to minimally affect insulin levels while avoiding potential off-target effects of random transgene insertion. Not so fast. Anecdotal evidence began to circulate that efficiency of gene deletion in these new lines could be low, particularly for the tamoxifen-inducible line. There was renewed speculation on the potential cause(s) of this new problem, potential reasons being the dose and/or method of tamoxifen administration, accessibility of the floxed target gene, or environmental influence from different mouse facilities. It seems that the field was faced, yet again, with another roadblock and a new mystery.

In the current issue of *Endocrinology*, Mosleh et al (8) shed light on this new issue by showing that the *Ins1* promoter is susceptible to hypermethylation, leading to genetic silencing of the locus and reducing Cre-recombinase expression. Testing across multiple research institutes, the authors illustrate problems with recombination efficiency at multiple floxed alleles (eg, *Creb*, *Foxm1*, *G6pc2*, and *Pcbp2*) both for the constitutive and inducible β cell Cre-driving lines. Some floxed genes show no recombination at all, despite demonstrating high recombination efficiency using reporter lines. When compared to other Cre lines (eg, *RIP*-Cre^25Mag^ and *MIP*-Cre/ERT^1Lphi^), the *Ins1*-Cre transgenes produce inconsistent and often milder phenotypes, and expression of Cre-recombinase in islets of these new lines was much lower. They attribute low Cre expression to increased methylation of CpG islands in the *Ins1* promoter and transgene locus and conclude that hypermethylation leads to insufficient transgene expression, preventing efficient recombination of floxed genes.

This study brings up important considerations for the field. There are now many mouse lines using a similar* Ins1* promoter knockin approach. Although targeting the endogenous *Ins1* locus avoids random insertion off-target effects, is silencing inevitable? Is methylation specific for the *Ins1* locus, or could it occur at all insulin genes? Decreased targeting efficiency is not yet reported for mouse lines using *Ins2* or *Pdx1* loci. Interestingly, recombination efficiency using the same mouse lines seems to differ greatly between research labs and across institutions. Perhaps methylation status is influenced by genetic background, housing conditions, diet, or other environmental factors, all of which may prove difficult to identify and control. One approach could be to mutate CpG islands in transgenes to prevent hypermethylation. But will this create new, unforeseen problems?

The β cell field seems cursed in its search for the best tools for in vivo gene targeting. It begs the question: Should we develop new models, or make the best of what we have? The *Ins1* promoter remains one of the best drivers of β cell–specific expression (2). The *Ins1*-Cre^Thor^ lines still work well for many laboratories and these mice remain the tool of choice because of high specificity and lack of hGH.

We believe that regardless of Cre driver chosen, including control groups expressing the Cre-transgene alone effectively controls for off-target effects. If recombination in other tissues is a concern, researchers could confirm β cell specificity of phenotypes using 2 separate Cre-drivers, *Pdx1* and *RIP* or *MIP*. These strategies, however; cannot overcome new challenges created by unpredictable and progressive transgene silencing. The current study by Mosleh and colleagues emphasizes the requirement to test (and retest) recombination efficiency at the targeted locus and not rely on reporter mice or past publications to claim effectiveness.

Although this may seem like a “β cell–world problem,” issues with LoxP/Cre technology are likely widespread. The Mosleh study emphasizes the need for careful experimental design of focused gene knockouts in all cell types and broadens the understanding of ectopic gene regulation. How different cells react to genetic manipulation in unique ways has implications for gene-targeting approaches both in basic science and medicine.

## Additional Information

***Disclosure Summary:*** The authors have nothing to disclose.

***Data Availability:*** Data sharing is not applicable to this article as no datasets were generated or analyzed during the current study.
